# Structure–Property Relationships in Polyethylene-Based Composites Filled with Biochar Derived from Waste Coffee Grounds

**DOI:** 10.3390/polym11081336

**Published:** 2019-08-12

**Authors:** Rossella Arrigo, Pravin Jagdale, Mattia Bartoli, Alberto Tagliaferro, Giulio Malucelli

**Affiliations:** 1 Department of Applied Science and Technology, and local INSTM Unit, Viale Teresa Michel 5, 15121 Alessandria, Italy; 2 Department of Applied Science and Technology, C.so Duca degli Abruzzi 24, 10129 Torino, Italy; 3 Italian Institute of Technology, Via Livorno 60, 10144 Torino, Italy

**Keywords:** polyethylene, biochar, rheological behavior, stress relaxation, thermo-oxidative stability

## Abstract

In this work, biochar (BC) derived from spent coffee grounds has been incorporated into high density polyethylene (PE) through melt mixing. The influence of the filler content on the rheological and thermal behavior of the obtained composites was assessed. In particular, a rheological study was performed systematically using different flow fields, including linear and nonlinear dynamic shear flow, revealing that the dynamics of PE macromolecules in the composite materials are slowed down because of the confinement of the polymer chains onto the filler surface and/or within the BC porous structure. Oscillatory amplitude sweep tests indicated that composites show weak strain overshoot behavior in the nonlinear regime: This finding clearly proves the formation of weak structural complexes, which cause a retardation of the macromolecular chains dynamics. Furthermore, the embedded BC particles were able to improve the thermo-oxidative stability of PE-based composites, remarkably increasing the PE decomposition temperatures.

## 1. Introduction

Increasing attention towards environmental safety and the consequent rising demand for new eco-friendly products, has stimulated the interest of both academic and industrial research to investigate renewable and bio-based materials, with the aim to develop a more sustainable manufacturing approach [1,2,3]. In this context, the use of natural fibers and particles for the production of polymer-based composites has received great importance [4,5]; in particular, bio-derived fibers, such as cotton [6], flax [7], or jute [8], have been embedded in several polymeric matrices, showing the potential to substitute the fillers traditionally used in polymer composites, also due to their advantageous price–volume–performance relationships [9,10]. However, because of the low thermal stability of natural fibers [11], their use in polymer-based composites has been often problematic; in fact, the degradation of bio-derived fillers occurs in the temperature range typical of polymer processing, and this feature limits the choice of the polymer matrix and significantly limits a successful processing of the composites [12,13]. Additionally, natural fibers show poor compatibility with most polymers, therefore chemical and/or physical treatments of their surface are often mandatory to enhance the polymer/filler interface [14,15]; lastly, a further drawback of bio-derived fibers refers to their high hydrophobicity which restricts their incorporation in moisture sensitive polymeric matrices [16].

A promising alternative to the traditional natural fibers is biochar (BC), a solid product obtained from the thermo-chemical conversion of biomasses in an oxygen-limited environment [17,18,19]. BC is a by-product of the bio-refinery industry [20,21,22] and it can be also obtained through pyrolysis from a variety of agricultural and forestry wastes [23,24,25]; it is a well-studied material in the field of soil amendment and contaminant absorption [26,27]. Recently, it has attracted considerable attention due to its intriguing characteristics: First, BC possesses high thermal and chemical stability, excellent electric conductivity, and a great surface area compared to natural fibers [28,29], notwithstanding its lower aspect ratio and poorer mechanical properties with respect to traditionally used fillers; besides, suitable pyrolysis conditions lead to uniform carbon-based and porous structures, potentially containing abundant functional groups, which could interact with polymer functionalities when BC is embedded into the host matrix [30]. All these features are increasingly making BC an attractive filler for improving mechanical, electrical, and physical properties of polymer composites [31]. For instance, BC derived from pine wood waste was introduced within a polypropylene matrix through a double-step processing, including melt mixing and injection molding [32]. The addition of increasing BC amounts progressively improved the tensile modulus and the flexural strength of resulting composites, which also showed enhanced thermal stability with respect to the neat matrix. Furthermore, composites based on epoxy resin and two different kinds of BC from maple tree pyrolyzed at two temperatures have been formulated, showing a brittle-to-ductile transition of the mechanical behavior in comparison to that of the unfilled resin [33]. Interestingly, BC has also been used to enhance the electrical conductivity and electromagnetic interference shielding performance of a blend of polyolefins [34]. Recently, BC derived from waste coffee grounds was investigated as a sensing material for developing sensors materials [35]. More specifically, the composite films, obtained by a screen-printing technique using polyvinyl butyral as a binder, showed high humidity sensitivity, fast response, and recovery times.

Though several studies report on the modification of the final properties of polymeric matrices through the introduction of BC, the effect of this filler on the polymer rheological behavior has not been systematically evaluated yet. The assessment of the viscoelastic response of polymer-based materials has a fundamental importance in the prediction of the final properties of polymer-based composites, providing a critical insight into the processing characteristics of these materials. In addition, the study of the rheological response of polymeric composites can be exploited for investigating their microstructure, offering the possibility to gain a fundamental understanding of their structure–properties relationships [36,37].

At present, Polyethylene (PE), due to its cost effectiveness and versatile characteristics, is broadly employed in a great variety of industrial applications, including automotive, domestic, furniture, packaging, among a few to mention [38,39]. However, due to the increasing interest towards environmental safety, different strategies have been developed and encouraged to enhance the sustainability of PE and PE-based composites. In this context, the utilization of additives derived from natural and bio-based sources represents an effective approach to reduce the environmental impact of PE-based systems, reducing the potential hazards linked with pollution [40].

The present study is aimed at studying the effect of BC particles derived from waste coffee grounds on the structure–properties relationships of PE-based composites. For this purpose, different amounts of BC, ranging from 1 to 7.5 wt%, were embedded within the polymer matrix through melt mixing, and the morphology, rheological, and thermal behavior of the resulting composite systems were thoroughly investigated. Furthermore, the thermal and thermo-oxidative stability of formulated materials was assessed and compared to that of unfilled PE.

## 2. Materials and Methods

### 2.1. Materials

In this work, a high density polyethylene (PE), trade name Lupolen 4261AG from Lyondell Basel, was used as polymer matrix. The sample has a melt flow rate (190 °C/21.6 kg) of 6.0 g/10 min, density of 0.945 g/cm^3^, melting temperature in the range 120–140 °C.

### 2.2. Preparation of Biochar from Waste Brewed Coffee

**Figure 1:**
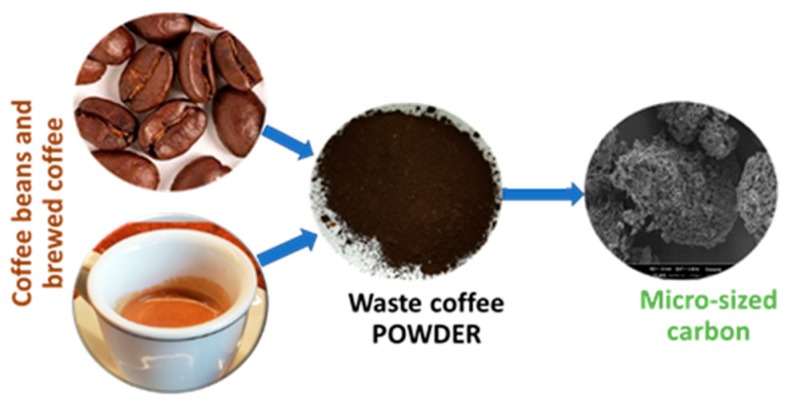
Extraction and conversion of waste coffee powder to carbonized biochar (BC).

A waste brewed coffee powder (Caffè Vergnano, Italy) was used as starting material for carbonization (Figure 1). Since the leftover chemicals in waste coffee powder after brewing may influence the natural structure of coffee during the carbonization step [41], in order to remove them the sampled waste brewed coffee powder was washed many times with demineralized water. The sample was further extracted by centrifugation followed by filtration. The waste brewed coffee powder was then dried in an oven at 90 ℃ for 10 h. The pyrolysis of the material was subsequently performed at 700 °C for 1 h in nitrogen atmosphere (gas flow: 120 mL/min). The heating ramp rate of the tubular furnace was set at 5 °C/min [42]. After the pyrolysis step, the material was manually ground in a mortar, achieving an averaged size of 10 microns [35].

### 2.3. Composite Preparation

The BC powder was embedded within the PE matrix by melt compounding, using a Brabender Plastograph mixer and operating at 220 °C, 100 rpm for 5 min. These conditions were optimized in order to avoid any degradation phenomena of the polymer matrix. Composites containing 1, 2.5, 5, and 7.5 wt% of BC were prepared. Unfilled PE was subjected to the same processing. Specimens for the rheological characterization were obtained by means of a compression molding step, using a laboratory press (Collin Teach Line 200T), working at 220 °C, under a pressure of 100 bar for 2 min.

### 2.4. Characterization

Microstructures were observed using a LEO-1450VP Scanning Electron Microscope SEM (beam voltage: 20 kV) on the nitrogen-fractured radial surfaces of the investigated samples. BC particles were observed using a field emission-scanning electron microscope (FE-SEM, Zeiss Supra-40, Oberkochen, Germany).

BC particle size distribution was evaluated using laser granulometry (Fritsch Analysette 22, Idar-Oberstein, Germany) after dispersion in ethanol through sonication in an ultrasonic bath for 10 min.

Pristine coffee and BC were analyzed through Fourier transform infrared (FT-IR) spectroscopy (Nicolet 5700, Thermoscientific) on attenuated total reflectance (ATR) mode (Smartorbit, Thermoscientific) in the range 500–4000 cm^−1^.

Rheological measurements were performed using an ARES (TA Instrument, USA) strain-controlled rheometer in parallel plate geometry (plate diameter: 25 mm). Strain sweep tests were carried out at 220 °C and ω = 1 rad/s. The complex viscosity and storage and loss moduli were measured performing frequency scans from 10^−1^ to 10^2^ rad/s at 220 °C. The strain amplitude was selected for each sample in order to fall in the linear viscoelastic region. Linear stress relaxation measurements were carried out at 220 °C, submitting the samples to a single step strain γ_0_ = 1%, and the shear stress evolution during time σ(t) was measured to obtain the relaxation modulus G(t) = σ(t)/γ_0_.

Differential scanning calorimetry (DSC) analyses were carried out using a QA1000 TA Instrument apparatus (Waters Lc, USA). All the experiments were performed under dry N_2_ gas (20 mL/min) using samples of around 8 mg in sealed aluminum pans. All the materials were subjected to the following cycle:

- First, heating up from 30 to 180 °C at 10 °C/min.
- Cooling down from 180 to 30 °C at 10 °C/min.
- Second heating up from 30 to 180 °C at 10 °C/min.

All the thermal parameters were evaluated on the second heating scan, erasing the previously thermal history and evaluating Tm (melting temperature) and Xc (crystallinity degree) in controlled conditions. Xc was calculated as the ratio between the heat of fusion of the sample, considering only the polymer fraction, and the heat of fusion of a 100% crystalline PE (293.6 J g^−1^ [43]).

Thermogravimetric analyses (TGA) were performed using a Pyris1TGA apparatus (Perkin Elmer, USA) (experimental error: ±0.5 wt%, ±1 °C). Samples (about 10 mg) were placed in alumina pans and runs were carried out in the range 50–600 °C, with a heating rate of 10 °C/min, under both N_2_ and air flow (35 and 25 mL min^-1^, respectively). T_5%_, T_10%_ (i.e., the temperatures at which 5% or 10% weight loss, respectively, occurs), and T_max_ values were calculated; besides, the final residue at 600 °C was measured.

## 3. Results and Discussion

### 3.1. Characterization of Biochar

**Figure 2:**
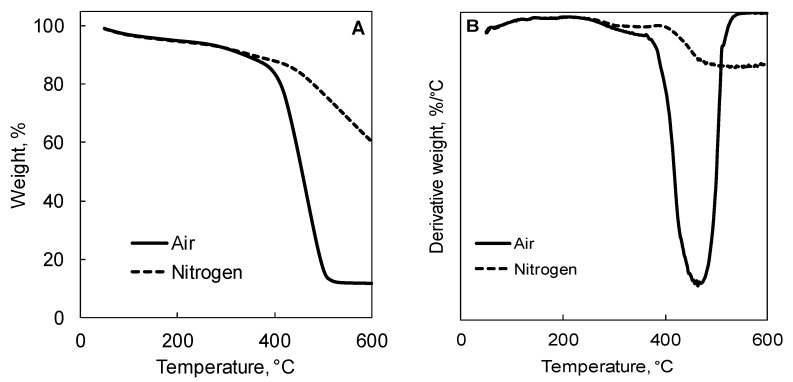
Termogravimetric (TG) (**A**) and dTG (**B**) curves in nitrogen and air of biochar.

Figure 2 shows the TG and dTG curves of BC. In an inert atmosphere, the degradation of biochar occurs in a single step (starting at about 400 °C), notwithstanding a limited weight loss between 80 and 120 °C, attributable to water removal; furthermore, a high residue (about 62%) is found at the end of the test (600 °C). A similar behavior is found also in air, where the degradation occurs in one step only, though at lower temperatures: The degradation is attributable to the oxidation of biochar, with the formation of CO and CO_2_. After the degradation step, a significant residue (about 11.9%) is found, indicating the quite high stability of the aromatic structure of BC.

**Figure 3:**
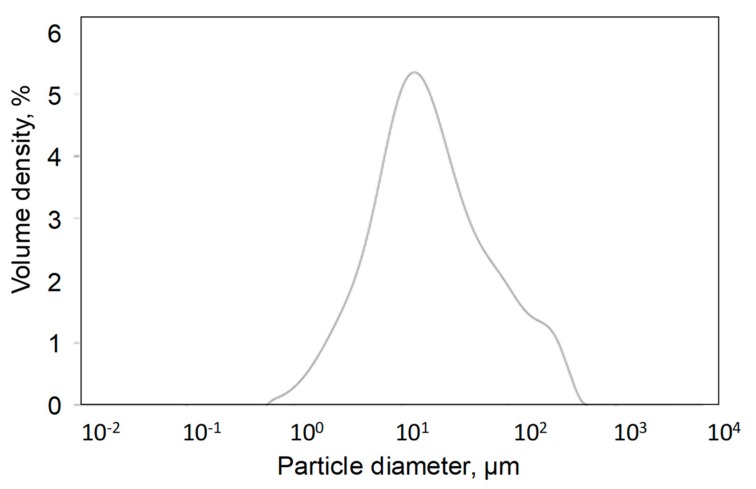
Particle size distribution of BC.

**Figure 4:**
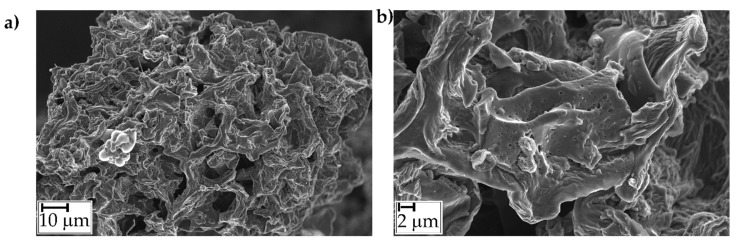
FE-SEM captures of BC at different magnifications.

BC was further analyzed through laser granulometry evaluating the particle size distribution shown in Figure 3. In particular, handily grounded BC shows a broad particle distribution with a major peak at around 10 μm and two shoulders at 100 μm and 500 μm, respectively. BC particles are characterized by a sponge-like shape as shown in Figure 4. Carbonaceous particles formed during pyrolysis show a diffuse large porous structure with an average pore size ranging from around 10 to 20 μm (Figure 4a). The inter-pore walls show a thickness of about 2 μm (Figure 4b) and the presence of tiny pores probably formed by the formation of volatile organic compounds during pyrolysis.

**Figure 5:**
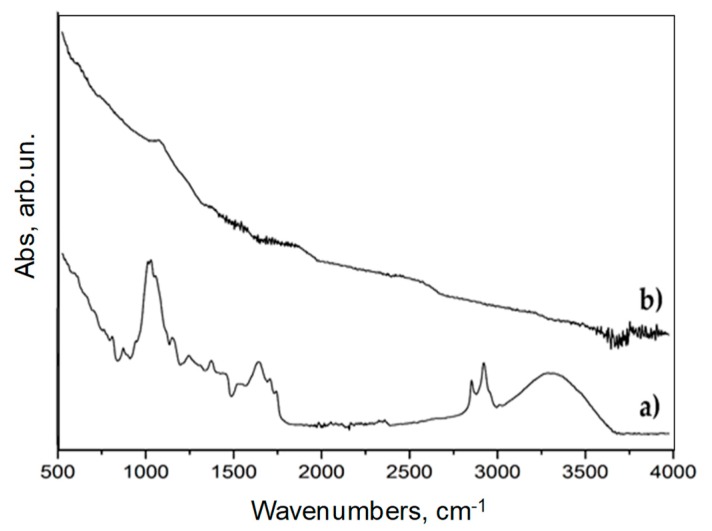
FT-IR (ATR mode) of (**a**) pristine coffee and (**b**) BC in the range from 500 to 4000 cm^−1^.

As shown by the FT-IR (ATR mode) spectra reported in Figure 5, coffee underwent a drastic carbonization process. Pristine coffee FT-IR spectrum (Figure 5a) shows a broad band due to ν_O–H_ at 3300–3600 cm^−1^ together with the signals of saturated symmetrical and asymmetrical ν_C–H_ at 2850–2950 cm^−1^. Furthermore, ν_C=O_ (1710–1741 cm^−1^) and ν_C=C_ at around 1540-1638 cm^−1^ due to the presence of aromatic structures are also detected. Several other signals (i.e., saturated and unsaturated δ_C-H_ at around 1370–1440 cm^−1^, saturated ν_C–C_ at 1243 cm^−1^) prove the massive presence of polysaccharides, aromatics, and organic molecules. All of those bands are not detected in the FT-IR spectrum of BC (Figure 5b). This is in accordance with the high pyrolytic temperature adopted for the carbonization of coffee residues. At 700 °C, all of the residual organic BC functionalities are almost completely removed and remain only as negligible traces.

### 3.2. Characterization PE-Based Composites

#### 3.2.1. Linear Rheological Behavior

**Figure 6:**
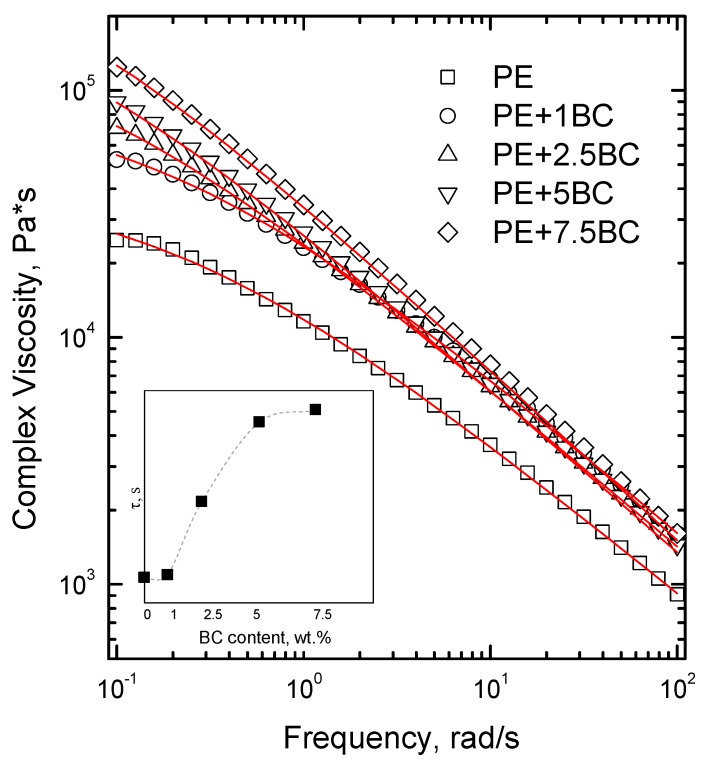
Complex viscosity as a function of frequency of neat Polyethylene (PE) and its composites (continuous lines correspond to cross model fit). In the inset, the variation of the relaxation time (τ) versus the content of BC is reported.

In Figure 6, the trends of complex viscosity as a function of frequency for the BC-containing composites are reported and compared to that of unfilled PE. The pure matrix essentially exhibits a power-law behavior, showing a weak plateauing of the complex viscosity only at the lowest investigated frequencies. The composite containing 1 wt% of BC shows higher viscosity values than neat PE, without a significant modification of the curve trend, according to what is usually reported in literature concerning the rheological response of polymer composites containing micrometric fillers [44]. Interestingly, composites with higher contents of BC exhibit a yield stress in the low frequency region that is progressively more pronounced with increasing the amount of filler. In general, this peculiar behavior is associated with the occurrence of polymer/polymer, polymer/filler, or filler/filler interactions, which can restrict the chain mobility causing an upturn of viscosity at low frequencies. To better investigate this phenomenon, the experimental viscosity data have been fitted using the Carreau–Yasuda model:

(1) $$\eta {\;}^{*}\dot{=\frac{\sigma_{0}}{\omega }+\;\eta_{0}{\left[1+{\left(\tau \omega \right)}^{a}\right]}^{\left(n-1\right)/a}}$$

where *σ*_0_ is the yield stress, *η*_0_ is the zero-shear viscosity, *τ* is the relaxation time, *a* is the Yasuda parameter, and *n* is the dimensionless power law index [45]. The value of the shear stress, as inferred by the trend of the complex viscosity curves, increases with increasing the BC content; furthermore, as shown in the inset of Figure 2, the relaxation time value increases as well. This finding indicates that the observed modification of the viscosity curve upon the addition of BC particles could be related to the restriction of the PE chain mobility as a result of some interactions taking place between polymer matrix and the embedded fillers.

**Figure 7:**
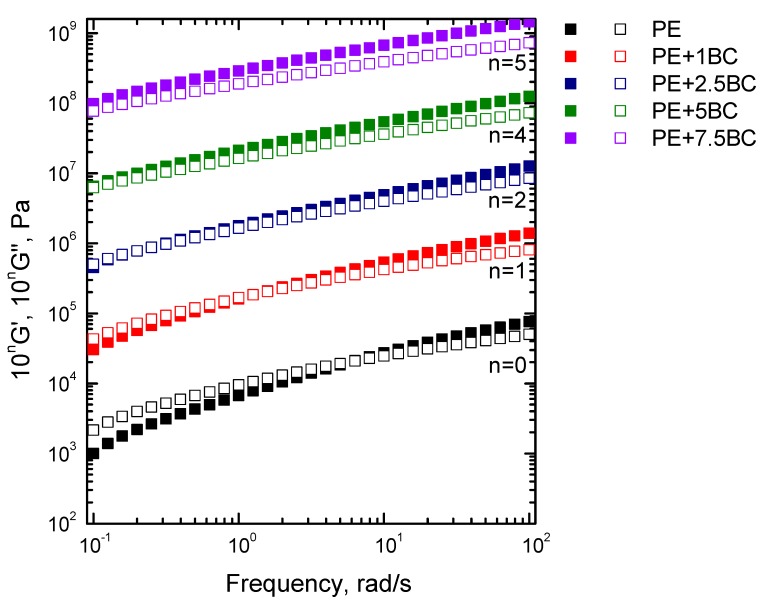
Storage (G’) and loss (G”) moduli for neat PE and BC-containing composites.

In Figure 7, the dynamic storage and loss moduli as a function of frequency are shown. The presence of increasing amounts of BC particles causes a progressive decrease of the terminal slope of the moduli curves in the low frequency range. Additionally, the cross-over frequency, i.e., the value of frequency, at which the dynamic moduli curves cross, shifts to lower frequency values with increasing the BC content, indicating a transition from liquid-like (G’(ω) < G”(ω)) to solid-like (G’(ω) > G”(ω)) rheological behavior. As already inferred from the analysis of the complex viscosity curves, the observed amplification of the elastic feature of the viscoelastic response of the composite systems can be related to the slowing down of the macromolecular chains dynamics. Noticed alterations of rheological behavior have been extensively documented in literature and have been assigned to incomplete relaxation of a percolation network formed by embedded particles [46] or of a polymer/filler hybrid structure [47]. In this last case, the macromolecules can be absorbed on the filler surface, forming an immobilized layer in the interfacial region; as a consequence, the motion of the polymer chains is slowed down or inhibited, and their relaxation requires longer time scales.

#### 3.2.2. Non-Linear Rheological Behavior

The modification of rheological behavior induced by the different interactions existing in polymer-based composites becomes more pronounced when the viscoelastic response of the material is tested under large deformations. For this reason, non-linear rheology can provide more information about the structural relaxation of a composite system, compared to conventional rheological measurements.

**Figure 8:**
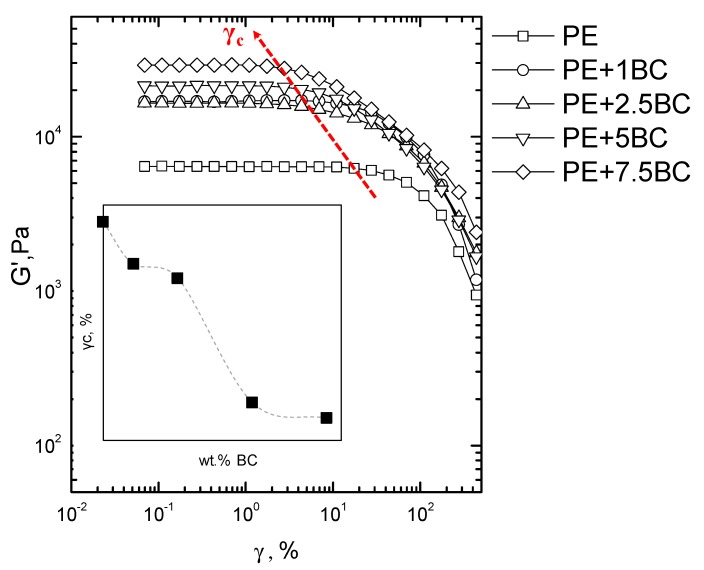
Storage modulus as a function of strain for neat PE and all BC-containing composites. In the inset the variation of the critical strain (γ_C_) versus the weight content of BC is reported.

Figure 8 shows the dynamic storage modulus (G’) of PE and PE-based composites, recorded during a strain sweep measurement. All investigated systems exhibit a plateau in the low strain amplitude region, followed by a dramatic drop of the modulus that marks the transition from the linear to nonlinear viscoelastic regime. This transition takes place when the value of G’ begins to decrease and it is due to the dissociation of the entanglements and to the orientation of chains along the flow direction [48]; more specifically, the value of strain, at which the modulus decreases more than 5% from its initial value, is called critical strain amplitude (γc).

From the curves collected in Figure 8 it is possible to note that the modulus value increases with increasing the BC amount, exhibiting the same trend already observed during frequency sweep tests; furthermore, the value of the critical strain amplitude, whose variation as a function of the BC amount is plotted in the inset in Figure 8, progressively decreases for the composites, indicating that the linear viscoelastic range is very sensitive to the particle content.

**Figure 9:**
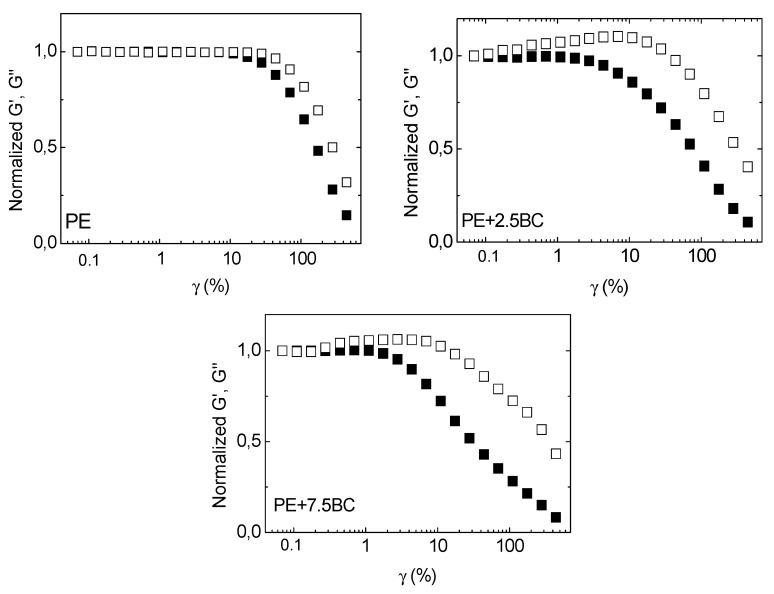
Normalized moduli (full symbols for G’ and empty symbols for G”) for neat PE and composites with 2.5 and 7.5 wt% of BC.

Figure 9 shows the normalized moduli response for unfilled PE and the composites containing 2.5 and 7.5 wt% of BC. PE shows the typical shear thinning behavior with a gradual decrease of both moduli as a function of particle content; conversely, BC-containing composites display a clear loss modulus overshoot behavior for both the filler loading levels. Usually, polymer-based systems show four different types of strain behavior: Shear thinning, strain hardening, weak and strong strain overshoots; the distinctive feature of these latter behaviors refers to a pronounced local maximum in the curve of G” or of both moduli, respectively [49]. In particular, the G” overshoot is indicative of formation of weak structural complexes that oppose to the imposed strain up to a certain deformation level, causing the increase of G”. Then, the formed structure is destroyed by deformation over the critical strain and polymer chains align along the flow direction, causing the decreasing of G” [50]. In PE/BC composites, the G” overshoot occurs in a time scale corresponding to the relaxation of polymer chain segments, suggesting the establishment of polymer-particle interactions that can hinder the motion of PE macromolecules, causing a retardation of their relaxation.

#### 3.2.3. Stress Relaxation Behavior

**Figure 10:**
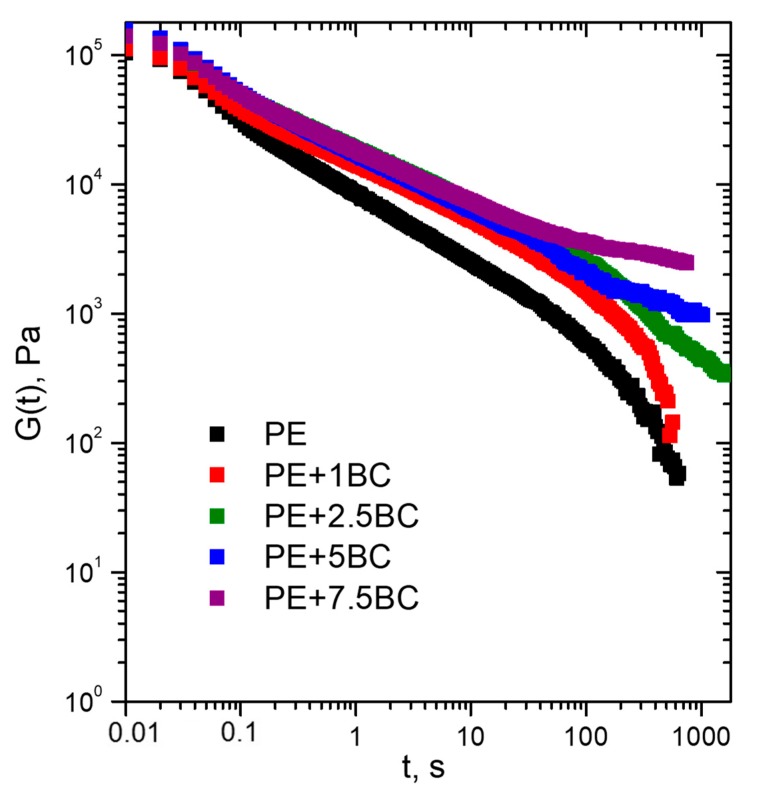
Stress relaxation curves for all the investigated systems.

The ability of the BC particles and of the polymer-BC interactions to affect the relaxation dynamics of PE chains has been confirmed by stress relaxation measurements. The shear relaxation moduli G(t) of neat matrix and BC-based composites are reported in Figure 10. At short times, the stress relaxation behavior is quite similar for both the composites and unfilled matrix; otherwise, at long times different relaxation kinetics can be observed. In particular, unfilled PE and the composite containing 1 wt% of BC relax like a liquid, reaching an equilibrium value equal to zero. Differently, G(t) does not exhibit any sign of drop in the composites containing higher content of BC, indicating a significant modification of the PE relaxation spectrum; more specifically, for the composites the stress relaxes to an equilibrium value rather than zero, and this feature can be associated with the occurrence of a pseudo-solid-like behavior due to the formation of a polymer-particle network that restricts the dynamics of PE macromolecular chains.

#### 3.2.4. Morphology

**Figure 11:**
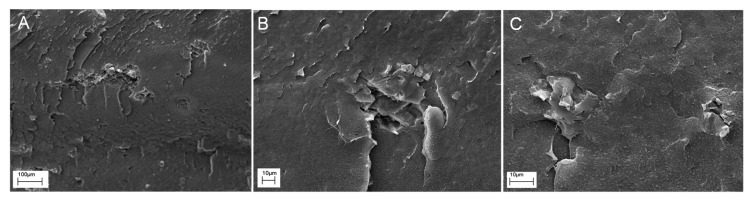
SEM micrographs of PE+2.5BC (**A**–**B**) and PE+7.5BC (**C**) composite at different magnifications.

Figure 11 shows typical SEM images of the fractured surface of composites containing 5 and 7.5 wt% of BC. The BC particles are homogeneously dispersed within the polymer matrix and do not tend to form aggregates; besides, it can be observed that the particles are firmly embedded within the polymer matrix, indicating a good matrix-filler adhesion. In addition, in the PE+7.5BC sample, the porous structure of BC is no longer observable, suggesting a partial filling of BC porosity by PE macromolecules, together with a partially disruption of particles with the formation of flakes from the original porous walls. These findings could have reasonably played a significant role in the modification of the rheological response of composite samples, since the confinement of the PE chains onto the surface of the embedded particles and the possible inclusion of the polymer chains within the BC channels can restrict the flow and the relaxation dynamics of PE macromolecules.

#### 3.2.5. Thermal Analyses

**Figure 12:**
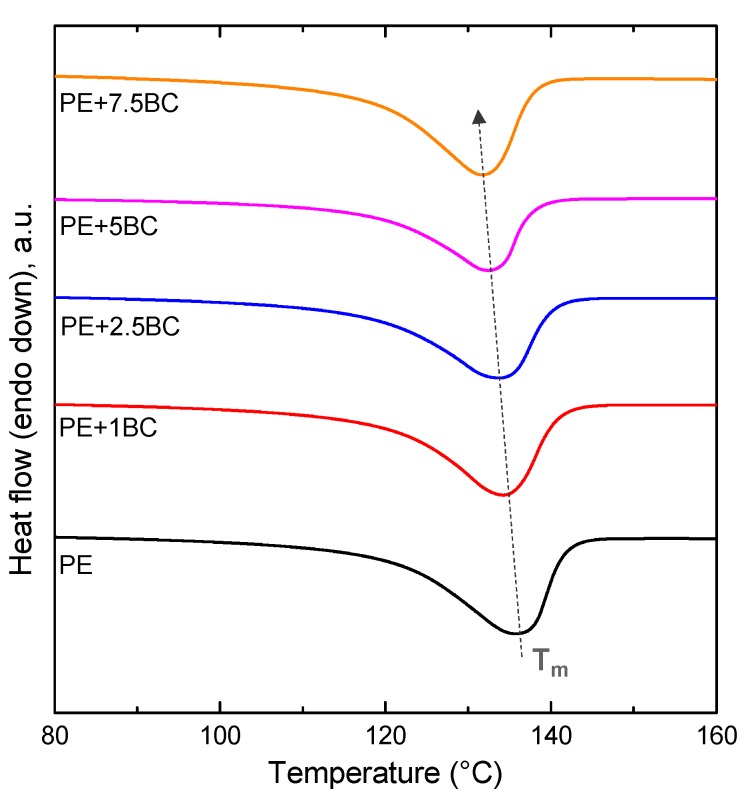
Thermograms (TG) curves recorded during the second heating scans for PE and its composites.

Figure 12 shows the TG curves for all investigated systems; the thermal properties resulting from the selected DSC cycle are collected in Table 1. First, it is worthy to note that the addition of BC particles induces an anticipation of the melting phenomena, causing a shift of Tm towards lower values; furthermore, a decrease of the crystallinity degree as a function of the BC content can be observed. This behavior can be explained considering some specific interactions occurring between the polymer chains and the particles interfaces. In fact, it is widely documented in literature that the immobilization of polymer chains onto the surface of micro- or nano-particles favors a reduction in the polymer crystallinity degree, since the portion of polymer in contact with the particle surface, being immobilized, is not able to form crystalline structures [31,51,52].

#### 3.2.6. Thermal and Thermo-Oxidative Stability

**Figure 13:**
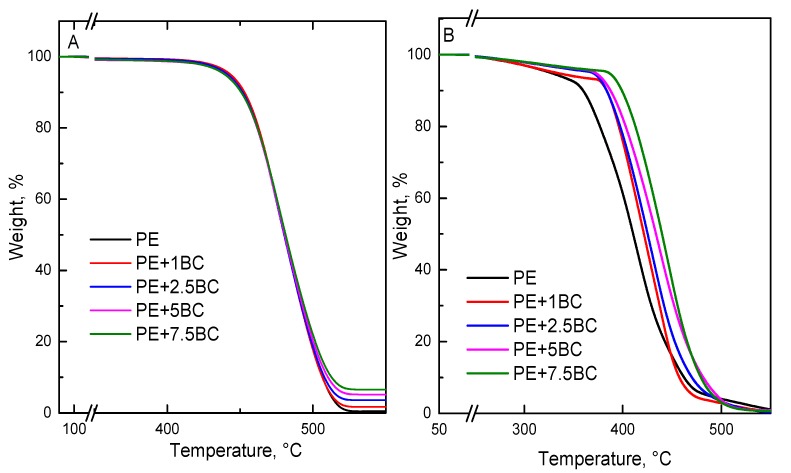
Thermogravimetric curves, either in N_2_ (**A**) or in air (**B**), for PE and all the investigated composites.

The thermal and thermo-oxidative stability of BC-containing composites was assessed through TGA analyses carried out in nitrogen and air, respectively. Table 2 collects T_5%_, T_10%_, T_max_, and the final residues at 600 °C in both the atmospheres for unfilled PE and the corresponding composites; the related curves are shown in Figure 13.

In nitrogen, the degradation of the composites occurs in a single degradation step, as clearly indicated by the TGA curves shown in Figure 9A. The presence of BC particles does not significantly affect the thermal stability of the polymer; in fact, the TGA curves of the composites show a trend very similar to that of the unfilled polymer, notwithstanding the final residue, which is related to the different BC content. In air, the presence of the embedded BC particles modifies the degradation mechanism of PE, with a remarkable delay of the onset of the decomposition. More specifically, both the initial degradation and the maximum degradation temperature of the composites increases with increasing the BC content, pointing out the beneficial effect of the embedded particles in enhancing the thermo-oxidative stability of the PE matrix. The increased thermo-oxidative stability could be attributed to the barrier effect exerted by the filler, which lowers the oxygen diffusion towards the degrading polymer matrix. As a matter of fact, as clearly shown in Figure 2, BC is stable at the temperature, at which polymer volatilization occurs. Furthermore, a carbonization effect of the PE matrix through an oxidative dehydrogenation cannot be excluded [53].

## 4. Conclusions

The effect of BC particles obtained from pyrolysis of spent coffee grounds on the rheological and thermal behavior of PE-based composites was investigated. An accurate rheological characterization was carried out, subjecting the neat matrix and the BC-containing composites to different flow fields, including linear and nonlinear dynamic shear flow. The obtained results indicated a slowing down of the relaxation dynamics of PE macromolecules, as a result of the confinement of the polymer chains onto the surface of the particles and/or within the BC empty channels. Stress relaxation measurements revealed a pseudo-solid-like behavior for the composites containing high amounts of BC, due to the formation of a polymer-particles network that restricts the dynamics of PE macromolecular chains. As probed by DSC characterization, the embedded particles were able to modify the melting enthalpy of the composites as compared to the neat matrix, inducing a decrease of the polymer crystallinity degree as well. Finally, thermogravimetric analyses confirmed that the presence of BC particles improves the thermo-oxidative stability of the composite systems, remarkably delaying the polymer degradation temperatures.

## Figures and Tables

**Table 1:** T_m_, ΔH_m_ and X_C_ values from Differential Scanning Calorimetry (DSC) analyses of PE and its composites.

Sample	T_m_ (°C)	ΔH_m_ (J/g)	X_C_ (%)
PE	138.4	209.7	71
PE+1BC	134.2	179.5	61
PE+2.5BC	133.8	177.3	60
PE+5BC	132.5	171.1	58
PE+7.5BC	131.7	164.5	56

**Table 2:** Thermogravimetric (TGA) results for neat PE and BC-containing composites.

Sample	T_5%_ (°C)	T_10%_ (°C)	T_Max_ (°C)	Residue at 600 °C (%)	T_5%_ (°C)	T_10%_ (°C)	T_Max_ (°C)	Residue at 600 °C (%)
	*N*_2_				*Air*			
PE	439.9	450.9	483.2	0.56	327.9	365.2	411.8	---
PE+1BC	443.0	452.5	482.1	1.65	330.4	383.3	422.3	0.06
PE+2.5BC	441.0	451.4	481.6	2.53	368.3	383.4	422.8	0.18
PE+5BC	439.1	450.8	481.7	5.08	371.7	387.4	426.5	0.63
PE+7.5BC	438.9	450.4	482.2	7.56	384.9	399.2	438.8	0.70
